# Detection of CTX-M-15 harboring *Escherichia coli* isolated from wild birds in Tunisia

**DOI:** 10.1186/s12866-018-1163-2

**Published:** 2018-04-02

**Authors:** Houssem Ben Yahia, Rym Ben Sallem, Ghassan Tayh, Naouel Klibi, Insaf Ben Amor, Haythem Gharsa, Abdellatif Boudabbous, Karim Ben Slama

**Affiliations:** 1 Laboratoire des Microorganismes et Biomolécules actives, Faculté des Sciences de Tunis, Université de Tunis El Manar, 2092 Tunis, Tunisie; 2 Institut Supérieur des Sciences Biologiques Appliquées de Tunis, Université de Tunis El Manar, 2092 Tunis, Tunisie

**Keywords:** *Escherichia coli*, ESBL, Molecular characterization, Integrons

## Abstract

**Background:**

The spreading of antibiotic resistant bacteria is becoming nowadays an alarming threat to human and animal health. There is increasing evidence showing that wild birds could significantly contribute to the transmission and spreading of drug-resistant bacteria. However, data for antimicrobial resistance in wild birds remain scarce, especially throughout Africa. The aims of this investigation were to analyze the prevalence of ESBL-producing *E. coli* in faecal samples of wild birds in Tunisia and to characterize the recovered isolates**.**

**Results:**

One hundred and eleven samples were inoculated on MacConkey agar plates supplemented with cefotaxime (2 μg/ml). ESBL-producing *E. coli* isolates were detected in 12 of 111 faecal samples (10.81%) and one isolate per sample was further characterized. β-lactamase detected genes were as follows: *bla*_CTX-M-15_ (8 isolates), *bla*_CTX-M-15_ + *bla*_TEM-1b_ (4 isolates). The IS*Ecp1* and *orf477* sequences were found respectively in the regions upstream and downstream of all *bla*_CTX-M-15_ genes. Seven different plasmid profiles were observed among the isolates. IncF (FII, FIA, FIB) and IncW replicons were identified in 11 CTX-M-15 producing isolates, and mostly, other replicons were also identified: IncHI2, IncA/C, IncP, IncI1 and IncX. All ESBL-producing *E. coli* isolates were integron positive and possessed “empty” integron structures with no inserted region of DNA. The following detected virulence genes were: (number of isolates in parentheses): *fimA* (ten); *papC* (seven); *aer* (five); *eae* (one); and *papGIII*, *hly*, *cnf*, and *bfp* (none). Molecular typing using pulsed-field gel electrophoresis and multilocus sequence typing showed a low genetic heterogeneity among the 12 ESBL-producing strains with five unrelated PFGE types and five different sequence types (STs) respectively. CTX-M-15-producing isolates were ascribed to phylogroup A (eleven isolates) and B2 (one isolate).

**Conclusion:**

To our knowledge, this study provides the first insight into the contribution of wild birds to the dynamics of ESBL-producing *E. coli* in Tunisia.

## Background

*Escherichia coli* (*E. coli*) is a facultatively anaerobic Gram-negative bacteria belonging to *Enterobacteriaceae* family [1]. *E. coli* is ubiquitous organisms that is found in soil, water, and vegetation and is part of the normal intestinal flora of human and animals. This intestinal bacterium acquires antimicrobial resistance faster than other conventional microorganism and is considered as a significant indicator for the selective pressure generated by antibiotic use [2, 3].

Extended spectrum β-lactamases (ESBL) are rapidly spreading in the last few years. CTX-M-15 is one of the most widespread ESBL types reported in many regions of the world. The association of *bla*_CTX-M-15_ gene with successful bacterial clones and epidemic plasmids is strongly implicated in its worldwide dissemination. CTX-M-15 production, which has been mainly identified in human and veterinary clinical samples, has occurred recently in wild animals, including wild birds, as well as in several environmental samples [4–15]. Besides resistance to extended-spectrum cephalosporins, ESBL-producing *E. coli* exhibit additional resistances to various antibiotics classified by the WHO as “critically important to human health” such as fluoroquinolones and aminoglycosides [16]. The spread of resistance to these antimicrobial classes in wildlife is therefore an alarming global health threat.

Tunisia is one of many countries with higher rates of bacterial pathogens with ESBLs-producers in the clinical setting, mostly associated with CTX-M-types [17]. Many previous studies have already been done by our research group about resistance mechanisms of *E. coli* isolates collected from different origins (human, animal, water and food chain), provided a great understanding of resistance evolution in different Tunisian environments. CTX-M-15 is the most predominant ESBL variant identified among clinical *E. coli* isolates in Tunisia, while CTX-M-1 enzymes has been identified in *E. coli* isolated from pets, food animals, healthy humans, foods, soil and water [6, 18–25]. Moreover, similarities between ESBL-producing *E. coli* collected from poultry, pets and human have been demonstrated by sequence typed results, highlighting the close connection between resistant isolates from different sources [19]. Although the occurrence and diversity of ESBL producing *E. coli* isolates from multiple sources in Tunisia has been well documented, data on antibiotic resistance in wild environmental reservoirs are limited. Furthermore, there is still a lack of information on the prevalence and characteristics of ESBLs producing *E. coli* isolates from wild birds in Africa. The purpose of this study was to evaluate the carriage level of ESBL-positive *E. coli* isolates in wild birds in Tunisia and to characterize their encoding genes in order to correlate data with previously obtained information from various sources in Tunisia.

## Methods

### Sample processing and susceptibility testing

One hundred eleven faecal samples were collected during March to mid-June 2012 from wild birds. Details of sampling including the establishment of the species origin of droppings and isolated sites, has been described in a last work [26], and summarized in Table 1. Avian fecal material (one per individual) were first cultured in Luria-Bertani (LB) broth for 16 to 20 h at 37 °C, 1 μl broth was then inoculated onto MacConkey agar plates supplemented with cefotaxime (CTX, 2 μg/mL). After incubation at 37 °C for 24 h, colonies showing *E. coli* morphology were recovered, identified by classical biochemical methods and by species-specific PCR (amplification of *uidA* gene) [25]. All cefotaxime-resistant *E. coli* isolates were screened for ESBL phenotype by double disk test [27]. One ESBL-producing *E. coli* isolate per positive sample was further characterized. Susceptibility to 17 antimicrobials agents was tested by disk-diffusion method [27]. Antibiotics tested were as follows: ampicillin, cefoxitin, ceftazidime, cefotaxime, imipenem, colistin, gentamicin, amikacin, tobramycin, streptomycin, nalidixic acid, ciprofloxacin, sulphonamides, trimethoprim-sulfamethoxazole, tetracycline, rifampicin, and chloramphenicol. *E. coli* ATCC 25922 was used as a control strain.

**Table 1:** Number and origin of faecal samples

Birds species		Number of faecal samples
Resident birds	European goldfinch (*Carduelis carduelis*)	17
	European greenfinch (*Carduelis chloris*)	35
	Pigeon (*Columba livia*)	7
	European serin (*Serinus serinus*)	7
Migratory birds	African river martin (*Pseudochelidon eurystomina*)	10
	Herring gull (*Larus argentatus*)	11
	Common blackbird (*Turdus merula*)	9
	Gray gull (*Leucophaeus modestus*)	7
	European bee-eater (*Merops apiaster*)	8

### Molecular typing and phylogenetic analysis of *E. coli* isolates

The clonal relationship among the cefotaxime resistant isolates was determined by pulsed-field gel electrophoresis (PFGE) using *Xba*I [28, 29]. All isolates were analyzed by MLST. Allelic profiles of these isolates were obtained after internal fragment sequencing of seven housekeeping genes (*adk*, *fumC*, *gyrB*, *icd*, *mdh*, *purA*, *recA*). Sequences Types (ST) were determined and compared with those included in the database (mlst.ucc.ie/mlst/dbs/ecoli) [30]. The isolates were allotted to the phylogenetic groups A, B1, B2, or D using a triplex PCR assay with specific primers for *chuA*, *yjaA* and TspE4.C2 markers as previously reported [31].

### Serotyping and virulence genotyping of *E. coli* isolates

All isolates were screened for O25b and O157 serotypes and for afa/dra operon [32, 33]. The detection of specific virulence factors including *stx*, *fimA*, *papG* allele III, *hlyA*, *cnf1*, *papC*, *aer*, *eae*, and *bfp* was performed by PCR. Primers and PCR conditions were used as previously described [34].

### Resistance genotype of *E. coli* isolates

DNA extraction was performed for all the strains by boiling. Specific detection of beta-lactamases genes was determined by PCR and sequencing for the following genes: *bla*_CTX-M_, *bla*_TEM_, *bla*_SHV_, *bla*_*OXA*_ and *bla*_CMY_. The genetic environments surrounding *bla*_CTX-M_ was characterized by PCR [35]. The presence of genes associated with resistance to tetracycline, sulphonamides, gentamicin and quinolones were determined as previously described [23, 29, 36–41]. Target genes and primers were summarized in Table 2. For the detection of chromosomal mutations, quinolone resistance-determining regions (QRDR) of the *gyrA* and *parC*, genes were amplified and sequenced. QRDR nucleotide sequences were compared with the reference sequences of the *E. coli* K-12 strain (GenBank accession no. U00096) [42]. Specific PCR was used to detect the *intI1* and *intI2* genes which encodes respectively for class 1 and class 2 integrases [29]. The variable regions of class 1 and class 2 integrons were characterized by PCR and sequencing in all *intI1*- or *intI2*-positive isolates. The presence of *qacE*∆*1*-*sul1* genes in the 3′-conserved segment of class 1 integrons was also investigated in all *intI1*-positive isolates [29, 43]. All PCR assays were performed with positive controls of the Microorganisms and Active Biomolecules Lab collection.

**Table 2:** Primers of the target antimicrobial resistance genes

Resistance genes	Primer sequence (5′-3′)	Size (pb)	Reference
*tetA*	F:GTAATTCTGAGCACTGTCGC	937	Sáenz, et al, 2004 [29]
	R:CTGCCTGGACAACATTGCTT		
*tetB*	F:CTCAGTATTCCAAGCCTTTG	416	
	R:CTAAGCACTTGTCTCCTGTT		
*sul1*	F:TGGTGACGGTGTTCGGCATTC	789	
	R:GCGAGGGTTTCCGAGAAGGTG		
*sul2*	F:CGGCATCGTCAACATAACC	722	
	R:GTGTGCGGATGAAGTCAG		
*sul3*	F:CATTCTAGAAAACAGTCGTAGTTCG	990	
	R:CATCTGCAGCTAACCTAGGGCTTTGGA		
*aac(3)-II*	F:ACTGTGATGGGATACGCGTC	237	Van de Klundert et al, 1993 [36]
	R:CTCCGTCAGCGTTTCAGCTA		
*aac(3)-IV*	F:CTTCAGGATGGCAAGTTGGT	286	
	R:TCATCTCGTTCTCCGCTCAT		
*qnrA*	F:AGAGGATTTCTCACGCCAGG	580	Cattoir et al, 2007 [37]
	R:TGCCAGGCACAGATCTTGAC		
*qnrB*	F:GATCGTGAAAGCCAGAAAGG	469	Wang et al, 2008 [39]
	R:ACGATGCCTGGTAGTTGTCC		
*qnrS*	F:GCAAGTTCATTGAACAGGGT	550	Cattoir et al, 2007 [37]
	R:TCTAAACCGTCGAGTTCGGCG		
*qepA*	F:GCAGGTCCAGCAGCGGGTAG	617	Rocha-Gracia et al, 2010 [38]
	R:GGACATCTACGGCTTCTTCG		
*aac(6′)-Ib*	F:TTGCGATGCTCTATGAGTGGCTA	482	
	R:CTCGAATGCCTGGCGTGTTT		
*gyrA*	F:TACACCGGTCAACATTGAGG	648	Oram and Fisher, 1991 [41]
	R:TTAATGATTGCCGCCGTCGG		
*parC*	F:AAACCTGTTCAGCGCCGCATT	395	Vila et al, 1996 [40]
	R:GTGGTGCCGTTAAGCAAA		

### Plasmid characterization

Plasmids from all strains were assigned to the incompatibility groups using PCR-based replicon typing (PBRT) [44].

## Results

Cefotaxime resistant *E. coli* isolates were detected in 12 out of 111 avian faecal materials analyzed (10.81%). The twelve fresh droppings were collected in Northern Tunisia and were recovered from the following free-living birds (number/area): *European serin* (five/ Bizerte), *Goldfinches* (two/ Bizerte), *Greenbirds* (two/ Bizerte), Harring Gulls (one/Bizerte), *Bee-eaters* (one/ Menzel Bouzelfa*)* and *Goldfinches* (one*/* Menzel Bouzelfa) (Table 3).

**Table 3:** Characteristics of the twelve extended-spectrum beta-lactamase (ESBL)-positive *Escherichia coli* isolates recovered from the faecal samples of birds

*E. coli* isolates	Species	Region of the sample	Virulence factors	Phylogroup	ESBL and genetic environment	Resistance phenotype to non-beta-lactam antibiotics	Other resistance genes detected	Replicons	PFGE	MLST
CbR33	*European serin*	Bizerte	*fimA-papC aer*	A	IS*Ecp1-bla*_CTX-M-15_-*orf477*	NAL-C-SXT-TET-STR-CIP	*tet(A), qnrA1, qnrB1, aac(6’)Ib-cr, aac(3)II*	HI2, W, FIC, FIB, P	P3	New ST^a^
CbR35	*European serin*	Bizerte	*fimA*-*eae*	A	IS*Ecp1-bla*_CTX-M-15_-*orf477*	NAL-C-SXT-TET-STR-CIP	*tet(A), qnrA1, qnrB1, aac(3)II*	HI2, W, FIC, FIB, P, A/C	P3	New ST^a^
CbR37	*European serin*	Bizerte	*fimA-papC*-*aer*	A	IS*Ecp1-bla*_CTX-M-15_-*orf477*	NAL-C-SXT-TET-STR-CIP	*bla*_TEM-1b_, *tet(A), qnrA1, qnrB1,* *aac(6’)Ib-cr, aac(3)II*	HI2, W, FIC, FIB, P, A/C	P3	New ST^a^
CbR38	*European Greenfinch*	Bizerte	*fimA-papC* *aer*	A	IS*Ecp1-bla*_CTX-M-15_-*orf477*	NAL-C-SXT-TET-STR-CIP	*tet(A), qnrA1, qnrB1, aac (6’)Ib-cr,aac(3)II*	HI2, W, FIC, FIB, P, A/C	P3	New ST^a^
CbR39	*European Goldfinch*	Bizerte	*fimA-papC*	A	IS*Ecp1-bla*_CTX-M-15_-*orf477*	NAL-C-SXT-TET-STR-CIP	*bla*_TEM-1b_, *tet(A), qnrA1, qnrB1, aac (6’)Ib-cr, aac(3)II*	HI2, W, FIC, FIB, P, A/C	P3	New ST^a^
CbR40	*European Greenfinch*	Bizerte	*fimA*	A	IS*Ecp1-bla*_CTX-M-15_-*orf477*	NAL-C-SXT-TET-STR-CIP	*bla*_TEM-1b_, *tet(A), qnrA1,qnrB1, aac(3)II*	HI2, W, FIC, FIB, P, A/C	P3	New ST^a^
CbR44	*Herring Gulls*	Bizerte	*fimA-papC*	A	IS*Ecp1-bla*_CTX-M-15_-*orf477*	NAL-C-SXT-TET-STR-CIP	*tet(A), qnrA1,qnrB1, aac (6’)Ib-cr, aac(3)II*	HI2, W, FIC, FIB, P, A/C	P3	New ST^a^
Cb11	*European Goldfinch*	Bizerte	*fimA-papC*	A	IS*Ecp1-bla*_CTX-M-15_ -*orf477*	NAL-C-SXT-TET-STR-CIP	*bla*_TEM-1b_, *sul3, tet(A), qnrA1, qnrB1, aac(3)II*	HI2, W, FIC, A/C	P3	New ST^a^
Cb36	*European serin*	Bizerte	*fimA* -*aer*	A	IS*Ecp1-bla*_CTX-M-15_ -*orf477*	NAL-C-SXT-TET-STR-CIP	*sul3*_,_ *tet(A), qnrB1, aac(3)II*	L/M, W	P2	ST 297
Cb37	*European serin*	Bizerte	*fimA-papC*-*aer*	A	IS*Ecp1-bla*_CTX-M-15_-*orf477*	NAL-C-SXT-TET-STR-CIP	*tet(A), qnrB1, aac (6’)Ib-cr, aac(3)II*	HI2, W, FIC, FIB, X, A/C	P4	New ST^b^
Cb62	*European Goldfinch*	Menzel Bouzalfa	*fimA*	A	IS*Ecp1-bla*_CTX-M-15_-*orf477*	–	–	I1,W, FIC, FIB,	P1	ST 410
Cb68	*European Bee-eaters*	Menzel Bouzalfa	*fimA*	B2	IS*Ecp1-bla*_CTX-M-15_-*orf477*	–	–	FIC	P5	ST 349

^a^Allelic combination: *adk* (6), *fumC*(4), *gyrB* (475), *icd* (New), *mdh* (454), *recA* (225), and *purA* (283)

^b^Allelic combination: *adk* (211), *fumC*(4), *gyrB* (4), *icd* (New), *mdh* (454), *recA*(225), and *purA* (283)

All CTX^R^ *E. coli* isolates exhibited an ESBL phenotype and expressed the CTX-M-15 enzyme. Four isolates co-expressed CTX-M-15 and TEM-1 enzymes.The IS*Ecp1* and *orf477* sequences were identified upstream and downstream of all *bla*_CTX-M-15_ genes.

All the twelve ESBL-positive isolates contained class 1 integrons with no inserted gene cassettes. No class 2 integrons was detected among the tested isolates. The CTX-M-15-positive *E. coli* also harbored genes encoding resistance to tetracycline [*tetA*], to quinolones [*qnrA*, *qnrB* and *aac*(6′)-Ib-cr], to sulphonamides [*sul3*] and to gentamicin [*aac*(3)-II].

The screening for Plasmid-Mediated Quinolone Resistance (PMQR) determinants among the 12 CTX-M-15-producing *E. coli* showed the presence of *qnrB1* + *qnrA1* + *aac-(6′)-Ib-cr* in 5 isolates, *qnrB1* + *qnrA1* genes in 3 isolates, *qnrB1* + *aac-(6′)-Ib-cr* in 1 isolate and *qnrB1* in 1 isolate. No mutations were found in the QRDRs of either DNA gyrase or topoisomerase.

Eleven of the twelve ESBL-positive isolates corresponded to the A phylogenetic group. The other isolate corresponded to the B2 group (Table 3).

Pulsed-field gel electrophoresis analysis demonstrated five different pulsotypes among the 12 ESBL-positive strains. Eight strains showed an identical PFGE pattern and were assigned to the same new sequence type (Table 3). These strains with an identical genetic backgrounds were recovered in the same sampling site from different free-living birds including *European serin* (*n* = 3), *Green birds* (*n* = 2), *Goldfinches* (n = 2), and *Harring Gulls* (*n* = 1). Three CTX-M-15-producing isolates had different PFGE profiles and were typed as ST297/A, ST410/A, and ST349/B2.

The *fimA* gene was the most commonly found among the 9 target virulence-associated genes and was detected in all strains. The *papC* and *aer* virulence genes were present in seven and five isolates respectively. The *eae* gene, detected in only one isolate, was found in conjunction with *fimA* gene. The 12 ESBL-producing isolates were negative for the remaining virulence or serotype traits tested.

A significant variability in plasmid profiles among the 12 *bla*_CTX-M-15_ *E. coli* isolates was observed and 7 different combinations have been shown (Table 3). IncF (FII, FIA, FIB) and IncW replicons were identified in 11 ESBL-producing strains, and in most cases, other replicons were also amplified: IncHI2 (9 strains), IncA/C (8 strains), IncP (7 strains) IncI1 (1 strain) and IncX (1 strain) (Table 3). The most prevalent replicon profiles identified were IncHI2 plus IncFIC plus IncFIB plus IncW plus Inc P plus IncA/C (six strains).

## Discussion

Our study is the first report of ESBL-producing *E. coli* with the *bla*_CTX-M-15_ gene in wildlife in Tunisia and Africa. These results show a high percentage of faecal carriage of ESBL-producing *E. coli* isolates from wild birds (10.81%) in samples obtained in 2012. Comparable prevalence was found in wild birds from various European countries where the detection rates varied between 8 to 16% [8, 45–47]. The first detection of ESBL-positive *E. coli* isolated from wild birds was reported since 2006 [48]. So far, Similar studies conducted in several European countries described a remarkable various percentage of ESBL carriage. Denmark and Poland were the least concerned countries with this phenomenon with (0%) and (0.7%) respectively. Spain (74.8%) and Netherlands (37.8%) constitutes the most two countries reporting high percentage of ESBL producing *E. coli* detected in wild birds, followed by Sweden (20.7%), Latvia (17.4%) and Portugal (12.7%) [12].

The carriage rate of ESBL-producing *E. coli* observed in this current investigation is higher than the incidence of ESBL-isolates detected in faecal samples of healthy humans in Tunisia [21]. These findings are in agreement with the Chilean study, which demonstrated a high prevalence of ESBL-positive *E. coli* among the gulls (30.1%) comparing to human population (12.2%) [10]. This illustrates that ESBL *E. coli* producers are common not only in humans or livestock farming but also in wild birds.

Genotypic characterisation of all ESBL- positive *E. coli* showed the detection of *bla*_CTX-M-15_ gene. Previous studies reported that this gene, widely described in *E. coli,* is implicated in nosocomial human infections worldwide. These data are in coherence with the human situation in Tunisia, where *bla*_CTX-M-15_ is the most detected genotype in human clinical settings while *bla*_CTX-M-1_ is most frequently detected among domestic animals [22, 24, 25]. This may suggest that the current carriage rate of ESBL *E. coli* producers found in wild bird is of anthropogenic nature. In the lack of other specific molecular data, we cannot exclude the possibility that the origin of the antibiotic resistance in some cases was not animal. It has indeed been recently shown that CTX-M-15 producers begin to appear outside of Tunisian hospitals and colonize animals [49, 50].

Besides the production of CTX-M-15, we detected several other resistance determinants, including genes encoding resistance for tetracycline [*tetA*], quinolones [*qnrA1*, *qnrB1*and *aac*(6′)-Ib-cr], sulphonamides [*sul3*] and gentamicin [*aac*(3)-II]. Given the fact that wild bird aren’t exposed to high doses of antibiotics, the resistance of their faecal flora is therefore directly acquired from their environment mirroring the dynamics of antimicrobial resistant bacteria in diverse ecological niches [11]. It is interesting to note that ESBL producing isolates recovered in the current study from free-living wild bird in urban area showed similar resistance phenotypes to those previously studied in human clinical settings in Tunisia. These facts probably indicate some human influence on the avian flora and might elucidate the acquisition and spread of antibiotic-resistant bacteria even without a direct antibiotic pressure [23]. Moreover, previous studies demonstrate that birds nesting near polluted waters harboured antibiotic-resistant *E. coli* with significantly higher frequencies than those associated with other environments. These results constitute a mirror of the potential role of human activity on the presence of antibiotic resistance genes in bird’s territory [51, 52].

Classification based on plasmid incompatibility revealed that CTX-M-15 producing isolates recovered in the present study harboured several plasmids belonging to major plasmid replicon types including IncF, I1, HI2, W, X, P, L/M and A/C. IncF plasmids (FIA, FIB, FIC, and FII) were the most prevalent replicon types and were detected in 11 strains (91.7%), similar to what has been reported in clinical data [53, 54]. In accordance to previous studies, we recorded an extensively distribution of the IncF replicons which seems to be well adapted to *E. coli* species [55, 56]. The *bla*_*CTX-M-15*_ gene has been frequently associated with IncF plasmids which could play a key role in promoting their rapid and global spread. [53, 56, 57].

The A/C replicon was detected in 8 isolates (66.7%), indeed this replicon has been considered to be widely common in *Enterobacteriaceae* and especially in *E. coli* isolates [58, 59].

Among the twelve CTX^R^ *E. coli*, ten isolates carried both of *bla*_CTX-M-15_ gene and PMQR genes, collected from five *European serin,* two *Green birds*, two *Goldfinches,* and one *Herring gulls*. These current findings showed that PMQR determinants were increasingly frequent in commensal isolates from wild birds in Tunisia. Previous studies emphasized that quinolone resistance is commonly associated to ESBL production highlighting that genes encoding resistance to beta-lactams and PMQR are frequently reported on the same mobile genetic element [6]. Interestingly, three PMQR determinants, *qnrB1*, *qnrA1* and *aac-(6′)-Ib-cr*, were detected in five different *E. coli* isolates. For all we know, the present study is the first report of these three PMQR determinants coexisting in an *E. coli* strain in Tunisia.

The enteropathogenic *E. coli* virulence factor *eae* was detected in one of the 12 CTX-M-15 producing isolates. It was isolated from *European serin*; this virulence gene was recently detected in a comparable frequency among ESBL-positive *E. coli* isolates of water samples in Tunisia [6].

The high prevalence of phylogenetic group A observed in this current study is in agreement with previous reports where commensal *E. coli* was found to be predominantly in phylogenetic groups A and B1 [21].

PFGE analysis highlighted similar macrorestriction patterns suggesting a clonal relationship for eight *E. coli* isolates; they were collected from four different bird species in the same city (Bizerte). Furthermore, Bizerte is a coastal city which combines several types of wild birds that shares the same source of water and feeding habits. It is likely that the eight multi-resistant CTX-M-15 harboring *E. coli* detected in this investigation is a result of clonal dissemination of successful clones even into an environment lacking antibiotic pressure.

The high carriage rate and modest genetic diversity of ESBLs producing *E. coli* from wild birds in Tunisia have also been reported in similar studies undertaken in The Netherlands, Spain and Germany, and might be explained by the existence of a common environmental source for the studied clonally related isolates [8, 15, 45]**.**

The ESBL-producing *E. coli.* STs present in avian fecal samples from this study included ST297, and ST349 appear to be common types in isolates from animal and environmental origin [60, 61]. Interestingly, the CTX-M-15-producing *Escherichia coli* isolates of lineages ST410-A identified in this study was previously reported in wildlife, humans, animals and environment [62–66]. This finding demonstrates successful transmission of clone ST410 *E. coli* between different hosts and ecosystems.

## Conclusion

To our knowledge, this study provides the first insight into the contribution of wild birds to the dynamics of ESBL-producing *E. coli* in Tunisia. Multidrug resistance bacteria transmission through avian mobility is still poorly understood and requires the implementation of several monitoring and control strategies.

## Abbreviations

CHL: Chloramphenicol
CIP: Ciprofloxacin
CTX: Cefotaxime
CTX^R^: Cefotaxime resistant
ESBL: Extended spectrum β-lactamase
MLST: Multilocus sequence typing
NAL: Nalidixic acid
PFGE: Pulsed-field gel electrophoresis
PMQR: Plasmid-mediated quinolone resistance
QRDR: Quinolone resistance-determining regions
STR: Streptomycin
SXT: Sulfamethoxazole-trimethoprim
TET: Tetracycline
